# Vascular Tissue Engineering: Challenges and Requirements for an Ideal Large Scale Blood Vessel

**DOI:** 10.3389/fbioe.2021.721843

**Published:** 2021-10-04

**Authors:** Chloé D. Devillard, Christophe A. Marquette

**Affiliations:** 3d.FAB, CNRS, INSA, Univ Lyon, CPE-Lyon, UMR5246, ICBMS, Université Lyon 1, Villeurbanne Cedex, France

**Keywords:** bioprinting, blood vessel, vascular tissue engineering, bioreactor, three-layered blood vessel, dynamic maturation

## Abstract

Since the emergence of regenerative medicine and tissue engineering more than half a century ago, one obstacle has persisted: the *in vitro* creation of large-scale vascular tissue (>1 cm^3^) to meet the clinical needs of viable tissue grafts but also for biological research applications. Considerable advancements in biofabrication have been made since Weinberg and Bell, in 1986, created the first blood vessel from collagen, endothelial cells, smooth muscle cells and fibroblasts. The synergistic combination of advances in fabrication methods, availability of cell source, biomaterials formulation and vascular tissue development, promises new strategies for the creation of autologous blood vessels, recapitulating biological functions, structural functions, but also the mechanical functions of a native blood vessel. In this review, the main technological advancements in bio-fabrication are discussed with a particular highlights on 3D bioprinting technologies. The choice of the main biomaterials and cell sources, the use of dynamic maturation systems such as bioreactors and the associated clinical trials will be detailed. The remaining challenges in this complex engineering field will finally be discussed.

## 1 Introduction

After many years of research in the field of engineering and regenerative medicine, an obstacle remains: the design and construction of vascularized large-scale *in vitro* tissues (>1 cm^3^) for biological studies and transplantation. As a result, the demand for blood vessels produced through bioengineering continues to grow and current options for vascular design remain limited. This makes this area a real challenge with advanced requirements such as the distribution, organization, adhesion, migration and maturation of living cells inside the constructs; the manufacturing or obtaining of a functional extracellular matrix (ECM); the development of a non-thrombogenic endothelium (Seifu et al., 2013).

A large blood vessel is organized as three concentric layers: the tunica intima, the media and the adventitia. These layers are composed of endothelial cells (ECs), smooth muscle cells (SMCs) and fibroblasts (FBs), respectively, trapped in a highly organized extracellular matrix (ECM) mainly composed of type I collagen, type III collagen and elastin.

The 1950s witnessed the appearance for the first time of tissue-engineering vascular grafts (TEVG) intended to replace occluded arterial vessels following surgical complications. They had the advantage of being recourse to the frequent shortage of allogenic transplants and made it possible to reduce the immunological rejection following transplants of large vessels of animal origin. However, despite advances in pharmacology, material science and device manufacturing, these synthetic TEVGs had not significantly reduced overall mortality and morbidity (Nugent and Edelman, 2003).

They were exclusively composed of acellular synthetic polymers and, as known today, the natural formation and maintenance of functional blood vessels is an active cell-driven process caused by the growth, death and migration of cells inside biomaterials, as well as by structural changes in tissues through the production and degradation of ECM by the cells themselves (Epstein et al., 1994; McGrath et al., 2005; Touyz, 2005).

Following these first steps, another approach emerged: the development of tissue-engineered vascular conduits. This technique was born from the growth of endothelial cells and/or smooth muscle cells, in extracellular matrix-like biomaterial components (Weinberg and Bell, 1985; Weinberg and Bell, 1986). However, the weak mechanical properties of the obtained vascular conduits, limited the applicability of the approach and demonstrated that the use of only one or two types of viable cells within a biomaterial matrix was not enough to recreate a viable blood vessel.

Then, the research in vascular tissue engineering focused on understanding the structural components of a blood vessel, increasing cells adhesion (van Wachem et al., 1985; McAuslan and Johnson, 1987; van Wachem et al., 1987; Absolom et al., 1988; McAuslan et al., 1988; Brunstedt et al., 1993; Wigod and Klitzman, 1993; Zhang et al., 1995; Cenni et al., 1996; Storck et al., 1996; Cenni et al., 1997) and tissue cohesion. These notions are vital for the rational design of biomaterials and the choice of an appropriate cellular source to give the blood vessel structural stability and facilitate its *in vivo* integration.

It is important to note that blood vessel size can vary considerably in the vascular hierarchy. The arteries and veins (large vessels with a diameter greater than 6 mm) are arranged for efficient transport over long distances while the capillaries (small vessels with a diameter less than 6 mm) are organized for optimal exchange of oxygen, nutrients and wastes. Therefore, it is not surprising that the requirements and design approaches for engineering large or small vessels are completely different. In the present review, we will only focus on the biofabrication of large vascular constructs. To sum up, the complex structures of blood vessels, the cellular sources and the required chemical signals make their construction a major challenge for the engineering of vascular tissues, while they shall present behaviors as close as possible to a natural ones. To reach this target, the engineered blood vessel must be biocompatible, degradable, have sufficient mechanical properties and permeability and being of variable size depending on the tissue or organ. To meet these requirements, different methods have emerged in recent years, such as sheet rolling, direct scaffolding, matrix molding and 3D bioprinting (Berman, 2012; Petrick and Simpson, 2013; Douglas, 2014; Schubert et al., 2014; Attaran, 2017). These approaches allow to mimic the necessary structure of large blood vessels for *in vitro* biological experiments and *in vivo* applications (Bajaj et al., 2014; Kang et al., 2016).

**FIGURE 1:**
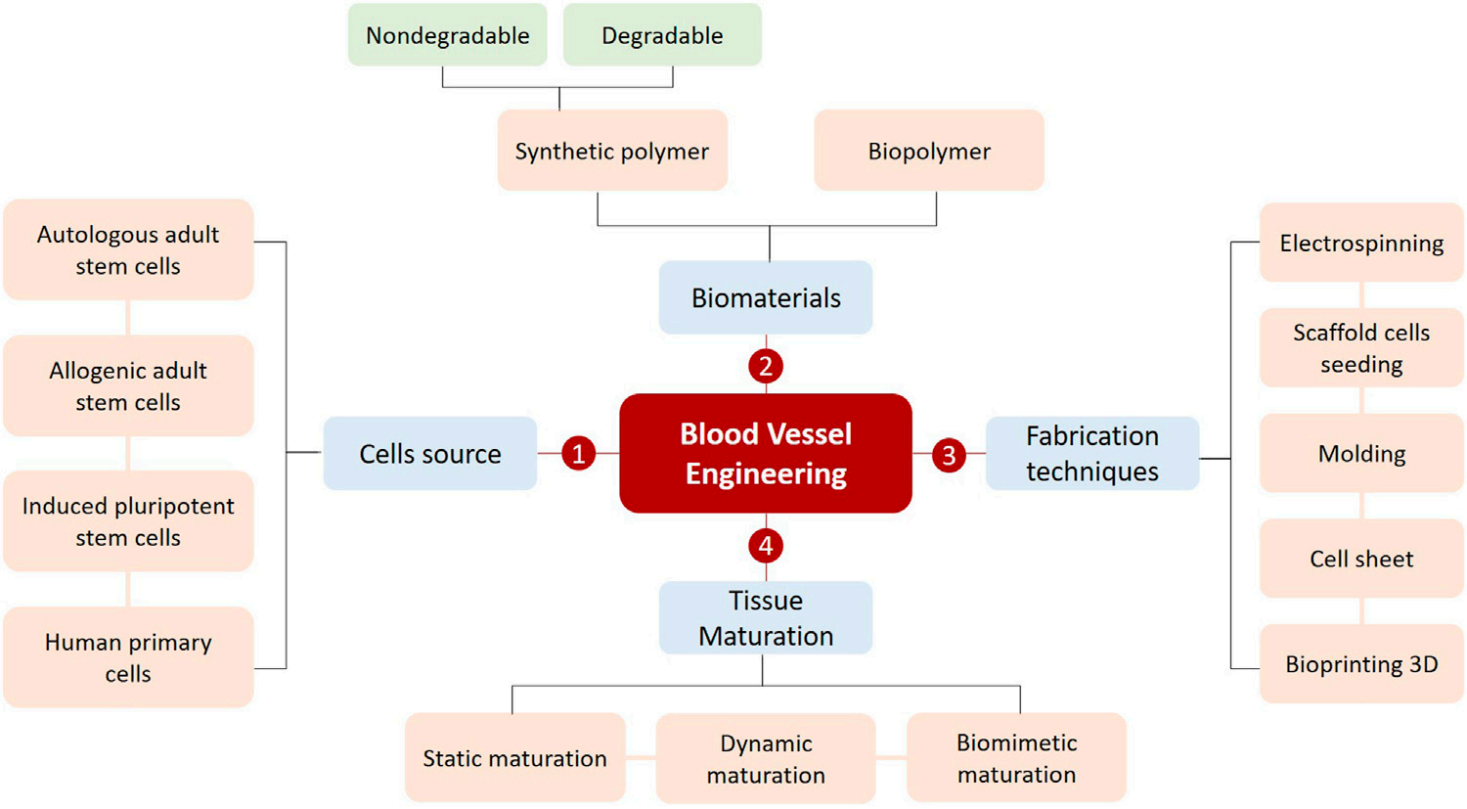
The fundamentals principles for successfully creating a blood vessel.

In this review, we will explore recent advances in bioengineering useful for the creation of large scale (>1 cm^3^), biologically functional blood vessels. We will highlight in particular the successful formation of multilayered blood vessels thanks to advanced manufacturing techniques which tend to become more and more efficient. Finally, we will present the remaining challenges of vascular tissue engineering as well as the potential solutions and future perspectives in this highly dynamic field of research (Figure 1).

## 2 Challenges and Requirements of an Ideal Large Blood Vessel

With several decades of exploratory and developmental studies, the field of vascular tissue engineering is advancing rapidly but is still facing numerous challenges. Indeed, when considering functional tissue replacement, mimicking defined characteristics of blood vessel is paramount and in order to design a long lasting, anatomically relevant blood vessel with biological functionality, it is crucial to take into consideration several requirements.

First, much of blood vessels structures and functions are derived from varying compositions at the biomolecular level. This is the reason why the structural components of the different vascular layers are essential to fully recapitulate vascular tissues. Understanding these elements is then mandatory for a rational conception and choice of biomaterials to be used in conjunction with appropriate cellular sources.

Secondly, the development of a biocompatible surface in contact with the blood is always a major challenge. Understanding and reproducing the surface in contact with the blood and its necessary anti-thrombogenic mechanisms will be essential to the successful realization of a blood vessel. Due to the complexity of the structure and composition of the blood vessel’s different layers, mimicking the surface in contact with blood remains a challenge. The use of specially designed biomaterials, cells and biological molecules (including pro-angiogenic factors) could help meet this challenge (Perets et al., 2003; Hegen et al., 2011; Nishiguchi et al., 2014; Poldervaart et al., 2014).

Then, the manufacturing technique involved must allow the generation of blood vessels with maximum cell survival and ideal recapitulation of the three essential vascular layers. Today, one of the biggest challenges in generating blood vessels is how to distribute the cells within the vascular tissue to recapitulate their natural arrangements (Rouwkema and Khademhosseini, 2016). In blood vessels, smooth muscle cells (SMCs) and endothelial cells (ECs) reside in the middle vascular layer (*tunica media*) and the inner vascular layer (*tunica intima*), respectively. When using acellular scaffold based approaches, the static method of seeding cells can only reach a suboptimal distribution since the SMCs cannot be located directly in the middle vascular layer and the ECs will not be homogeneously distributed on the luminal vascular layer. In addition, despite the integration of biological molecules such as collagen, fibronectin and cell adhesion peptides (RGD, fibronectin heparin binding sites, etc.) on the scaffold surfaces (Pawlowski et al., 2004; Sagnella et al., 2005), the cell seeding static method is not sufficient to obtain the trilayered structure of vascular tissue.

Finally, blood vessels must have sufficient mechanical properties to stand stitching during *in vivo* implantation and to support physiological blood pressure of approximately 2,000–3,000 mmHg (Yow et al., 2006; Konig et al., 2009). It is then now clear that producing cellularized tube-like structures recapitulating the composition of blood vessels might not be enough and that these structures shall be conditioned/matured within dynamic bioreactors. These active maturation conditions will be the final key to obtain functional implantable blood vessels.

## 3 Anatomy of Vascular Tissue

Blood vessels form compact closed circulatory systems that infiltrate most of the body’s tissues. Based on their structure and function, blood vessels are classified as either arteries, capillaries or veins (Tennant and McGeachie, 1990) The main function of the large vessels (arteries and veins) is to provide efficient transport to distant sites, while the small vessels (capillaries and arterioles) allow an optimal exchange of nutrients, oxygen and waste within organs and tissues. As a result, the design requirements and approaches for engineering large vessels (>6 mm) are completely different from those for manufacturing small vessels (<1 mm).

**FIGURE 2:**
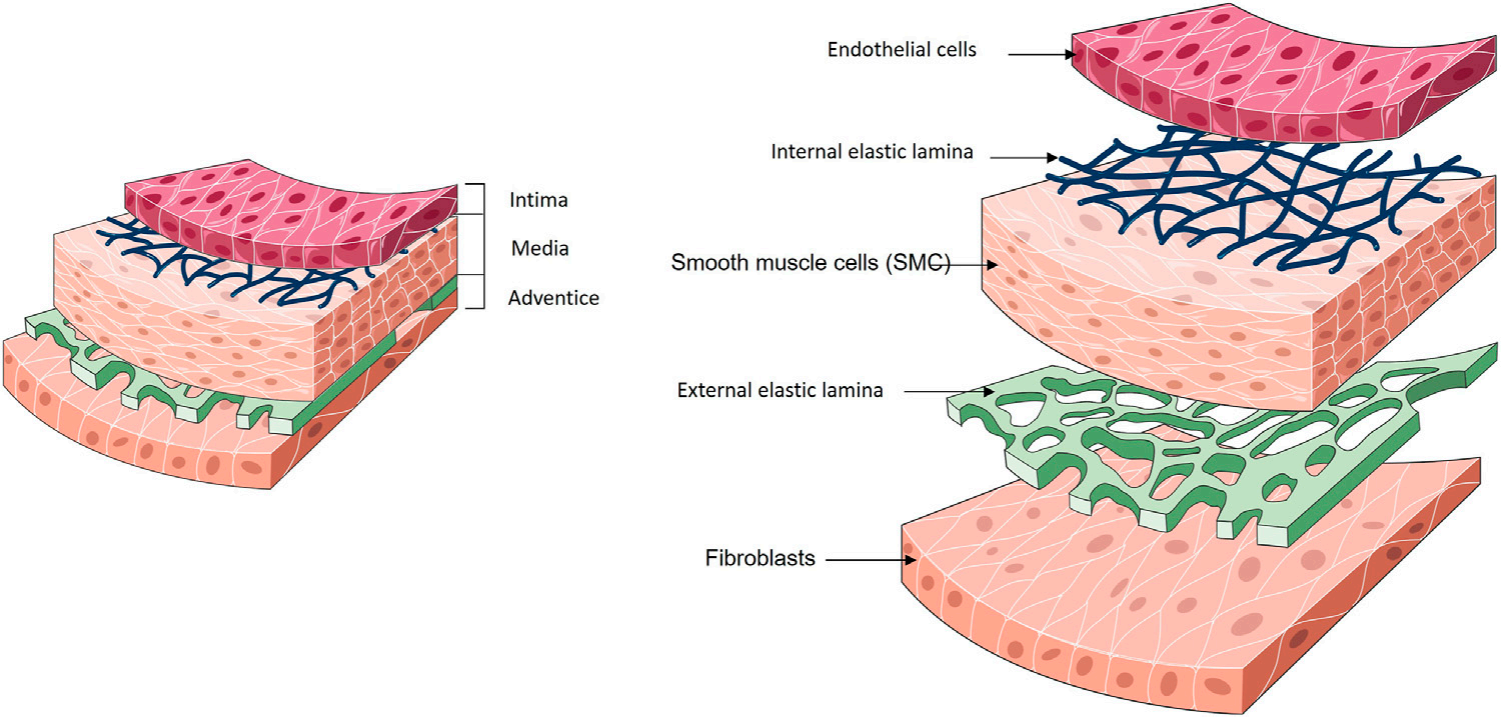
General structure of a blood vessel.

Upon these different types of blood vessels, arteries are the largest and most robust ones and their reinforcement/replacement is often necessary to restore healthy blood flow. To fulfil their physiological functions, arteries have a concentric three-layer structure where each layer is responsible for an independent but essential function: the *intima*, responsible for the anti-thrombogenic aspect; the *media*, responsible for mechanical strength; and the *adventitia*, a collagen-rich connective tissue which maintains the structure, otherwise the blood vessel would be weak and fragile (Figure 2).

### 3.1 Intima

The *intima* consists of an endothelium, a basal lamina and a cell-free sub-endothelial space. The endothelial cells (EC) monolayer of the *intima* forms a tight barrier between the lumen of the vessel and the vessel wall (Carabasi and Svigals, 2001). The ECs are in direct contact with the blood. ECs play then key roles in biological processes such as coagulation, inflammation, barrier function, blood flow regulation and synthesis/degradation of ECM components (Cines et al., 1998; Fauvel-Lafève, 1999).

The *intima* is separated from the *media* by a thin layer of elastin and type IV collagen, described as the internal elastic lamina (IEL) (Tucker and Mahajan, 2018). The IEL is porous and presents various pore densities depending on the region of the vascular tree (Stegemann et al., 2007). The primary function of the internal elastic *lamina* is linked to the elastic resilience of the vascular wall in order to maintain blood pressure, although recent experimental observations assign a multifunctional role for this structure. The fenestrations inside the IEL allow the diffusion of the nutrients from the vessel lumen towards the under-adjacent tissues of the vascular wall. It is a route by which the SMCs manage to penetrate the *intima*, but also a path to pathological status formation (Clowes and Schwartz, 1985; Ross, 1986).

### 3.2 Media

The *media* is composed of smooth muscle cells (SMC), type I and type III collagen (Berillis, 2013). These cells have a contractile phenotype which allows a contraction or dilatation of the vessels (Campbell and Campbell, 1985; Campbell et al., 1988; Berillis, 2013). The SMCs and collagen fibers are organized in a concentrically along the axis of the vessel.

### 3.3 Adventice

The outermost layer of the blood vessel wall is the adventice. It is composed of fibroblasts embedded in a loose collagen matrix. The adventice it is mainly composed of connective tissue like type I and type II collagen and prevents the vessel from over-extending or over-retracting. The mechanical properties of a blood vessel such as tensile strength are provided by the presence of these collagen fibers (Tennant and McGeachie, 1990). In the large arteries calibers, an external elastic lamina is observed between the *media* and the adventice.

It is then obvious that the blood vessels, even if considered as simple tubing, present a high complexity with different and specific layers interacting continuously with each other in order to enable remodeling and perfusion of the organs they irrigate. This complexity must be taken into account for the *in vitro* recapitulation of a blood vessel (Zhou and Tian, 2015).

## 4 Cells

An appropriate cell source is also essential for the reproduction of living vascular tissue, to provide structural stability and finally to facilitate *in vivo* integration.

The major limitations observed in the use of vascular cells are the source and quantity of cells. A vital factor to take into account is the proliferative potential of the cell, which decreases with the age of the donor but also varies according to the chosen cell type (Janzen et al., 2006; Mimeault and Batra, 2009).

First, autologous cells derived from patients are a potential cell source that has sparked interest because of their potential to minimize transplant rejection. However, the isolation and expansion of viable and qualified (according to regulatory affairs) primary cells to a therapeutically relevant scale may be a real bottleneck.

With the advancement of stem cell (SC) (Bajpai and Andreadis, 2012) technology, autologous and induced pluripotent stem cells (iPSC) (Hirschi et al., 2014) are emerging as promising alternative sources of EC and SMC (Chang and Niklason, 2017; Wang Y. et al., 2017; Chan et al., 2018).

Adult stem cells have the advantage of being used immediately after isolation, but their viability highly depends upon the patient’s health and age, leading then to complex therapeutic strategies and validations. This is the reason why embryonic stem cells (ESC) are now increasingly used for vascular tissue engineering studies. Nevertheless when it comes to clinical use, ethical questions and potential tumorigenicity problems arise.

Finally, iPSCs have the advantages of a quasi-unlimited quantity of available cells and possible differentiations to vascular-specific lineage (Luo et al., 2021; Nelson et al., 2021). However, all cell sources have their own limitations and just like ESC, iPSC raise concerns about the fate of the implanted cells and their possible role in tumorigenesis (Kanemura et al., 2014; Itakura et al., 2017; Takei et al., 2020). In addition, each research laboratory has its own differentiation protocols and it seems obvious that before using these differentiated cells for clinical applications, a reliable and efficient protocol demonstrating the effects of each differentiation step on the functionality of the obtained cells, must be established.

## 5 Biomaterials

An important factor to consider in vascular tissue engineering is the choice of biomaterials that will be associated with the cells to form the vascular tissue. As the architecture of the different vascular walls is complex, the selection of biomaterials is not obvious and requires careful consideration and requirements. This choice shall be based on mechanical, physical, chemical and biological properties, biocompatibility but also degradability. Important knowledges to be considered are also the biomaterial response to mechanical stimuli and temperature, their chemical composition and microstructure changes, together with their dynamics (Peppas and Langer, 1994; Hubbell, 1995; Vacanti and Langer, 1999; Hench and Polak, 2002; Bohner, 2010; Bas et al., 2018; Sharma et al., 2018). Today, a gap has formed between the rapid progress of tissue engineering techniques and biomaterials used for the reproduction of tissues and organs.

Many studies still use a single biomaterial to recapitulate an *in vitro* tissue. However, in the majority of cases, the presence of this unique biomaterial is not sufficient for cell survival and to ultimately reproduce viable tissue. In vascular tissue engineering, three classes of biomaterials are mainly used: synthetic polymers, biodegradable polymers and biopolymers (Table 1).

**TABLE 1:** The different categories of biomaterials used in vascular tissue engineering.

Biomaterial		Cell type	Biofabrication process	*In vivo* system	Culture time	References
Synthetic scaffold–*ex vivo* applications	ePTFE + fibrin coating	Human autologous ECs	Rotational seeding	Human	7 years	Meinhart et al. (2001)
	ePTFE + fibronectin coating	Autologous ECs seeded on SMCs	Rotational seeding	Rabbit	100 days	Yu et al. (2001)
	Dacron® + cells + veinous blood	Bone marrow derived CD34 cells	Static seeding	Canine	4 weeks	Bhattacharya et al. (2000)
Synthetic scaffold–*in vivo* applications	ePTFE + fibrin coating + FGF-1	—	—	Canine	140 days	Gray et al. (1994)
	Dacron®	—	—	Canine	2 weeks	Herring et al. (1979)
	ePTFE + VEGF	—	—	Rat	30 days	Randone et al. (2005)
	polyurethane + FGF—2 + heparin + gelatin	—	—	Rat	4 weeks	Doi and Matsuda (1997)
Biodegradable scaffold	Lycra + PEG + PLA	—	—	Canine	3 months	Izhar et al. (2001)
	PLGA + collagen	—	—	Canine	6 months	Iwai et al. (2004)
	PCL	—	—	Rat	24 weeks	Pektok et al. (2008)
	PLLA	Bone marrow MSCs	Cell sheet rolling	Rat	60 days	Hashi et al. (2007)
	PGA	Bovine SMCs	Static seeding	Bioreactor	4 weeks	Niklason et al. (1999)
	PGA	Lamb fibroblasts + ECs	Static seeding	Lamb	100 weeks	Hoerstrup et al. (2006)
Biopolymers	Type I collagen	Bovine fibroblasts, SMCs and ECs	Fibroblasts and SMCs molding, ECs seeding	—	4 weeks	Weinberg and Bell (1986)
	ECM cell production	Human fibroblasts, SMCs, ECs	Cell sheet rolling	Canine	1 week	L’Heureux et al. (1998)
	Fibrin	Rat SMC, ECs	Molding	—	6 days	Cummings et al. (2004)
	Xenogeneic extracellular matrix	human adipose-derived stem cells (hASCs) and normal human dermal fibroblasts (NHDFs)	Static seeding	Rat	6 weeks	Choi et al. (2012)
	Elastin	Human SMCs	Static seeding	—	14 days	Buijtenhuijs et al. (2004)
	Agarose-based gel	Human SMCs, ECs, NIH 3T3 fibroblasts	3D Bioprinting	—	10 days	Ozler et al. (2017)
	Alginate	Human SMCs, ECs	3D Bioprinting	Rat	3 weeks	Gao et al. (2019)
	Gelatin	SMCs	3D bioprinting	—	7 days	Tijore et al. (2018)
	Alginate/platelet-rich plasma (PRP)	HUVECs	3D bioprinting	—	3 days	Faramarzi et al. (2018)
	Alginate/gelatin	SMCs	3D Bioprinting	—	7 days	Duan et al. (2013)
	Gelatin/Fibrinogen	Human bone marrow MSC, Human fibroblasts, HUVECs	3D bioprinting	—	6 weeks	Kolesky et al. (2016)

Synthetic polymers, such as polytetrafluoroethylene (ePTFE), polyethylene terephthalate (Dacron®) and polyurethane have been widely studied and used since the early days of vascular engineering to create vascular substitutes (Kannan et al., 2005). However, the major bias in their use was their biologically inappropriate behavior at the blood interface. These synthetic polymers were for the most part of low permeability and did not allowed appropriate nutrients diffusion.

To overcome this limitation, formulation of different biomaterials has emerged, coupled with the seeding of these synthetic graft with vascular cells is then initiated (Herring et al., 1979). These studies were followed by the development of vascular constructs using bioactive materials such as collagen seeded with vascular cells (Weinberg and Bell, 1986). Finally, the direct use of extracellular matrix secreted by cells was used and associated with vascular cells to rebuild a blood vessel (L’Heureux et al., 1998) in which permeability and biocompatibility were restored. However, an obstacle persists: the mechanical resistance of the blood vessel that shall, once implanted, withstand mechanical pressures of up to 3,000 mmHg for the human artery (Yow et al., 2006). The ideal balance between adequate mechanical strength and the preferential use of natural biomaterials remains thus a challenge.

Most of the research in tissue engineering has been based on hydrogels composed mainly of a unique polymer such as alginate, gelatin, fibrin or agarose (Sahni and Francis, 2000; Wu et al., 2007; Garvin et al., 2011; Ceccarelli and Putnam, 2014; Jia et al., 2014; Kinoshita et al., 2016; Klotz et al., 2016). Nevertheless, a wider variety of hydrogels exist and (Billiet et al., 2012; Malda et al., 2013) research is now focused on the development of complex, multifunctional biomaterials encapsulating proteins of interest to guide the formation of vascular tissue. The challenge will be to find the right ratio between easily degrading biomaterials and at the same time, production of extracellular matrix by human cells, in a sufficient amount to replace and recreate the properties of biomaterials in a natural way.

Complementary studies on biomaterials were also performed to implement the use of synthetic materials with specific surface chemical modifications, with additions of specific coatings or even with modifications of proteins (Greisler et al., 1987; Gray et al., 1994; Greisler et al., 1996; Walluscheck et al., 1996; Meinhart et al., 2001; Nishibe et al., 2001; Krijgsman et al., 2002; Li et al., 2005; Randone et al., 2005).

Each of these strategies will have an impact on the final use of the blood vessel. If the research is focused on the *in situ* regeneration of the blood vessel, the choice of the material and its design must have as main objective the reaction to the immune system and therefore allows to initiate a natural healing response once the vascular graft implemented. If the research is based on the development of the blood vessel *ex vivo*, a step of *in vitro* culture of human vascular cells and an association of these with specific biomaterials allowing their adhesion, proliferation and maturation is vital. These steps of *in vitro* cell culture on suitable biomaterials will ultimately define the mechanical and biological properties of the blood vessel before its implantation.

## 6 Biofabrication Techniques

Another major factor in the formation of a physiologically functional blood vessel architecture are the manufacturing techniques employed. These techniques are numerous but can be segmented in two main strategies, 1) manufacturing of scaffolds subsequently seeded with cells and 2) fabrication of the structure directly in the presence of cells, including bioprinting.

### 6.1 Early Tissue-Engineered Blood Vessel

Since the 1950s, vascular grafts created from synthetic materials, therefore completely acellular, are commonly used in clinics to replace arteries with large diameters only (>6 mm) and the creation of vascular grafts with smaller diameters (<6 mm) led to many complications and are still challenging.

As a result, the emergence of Tissue-Engineering Blood Vessel (TEBV) was born. They are often formed from a collagen scaffold and are seeded with cells to reproduce the three vascular layers. This is the Weinberg and Bell project. The two researchers performed the first TEBV (Weinberg and Bell, 1986), a collagen scaffold on which they seeded fibroblasts for the adventice, SMCs for the *media* and endothelial cells for the *intima*. The weakness of this model was its low mechanical resistance: 120–180 mmHg were obtained for this TEBV compared to the expected 2,000 mmHg for the saphenous vein and 3,000 mmHg for the mammary artery in humans (Yow et al., 2006). However, this research has demonstrated the feasibility of performing a transplant of blood vessels developed by tissue engineering using human cells.

From this point, the idea of merging the mechanical resistance properties of vascular grafts with the physiological properties of TEBVs was born. In 1979, Herring et al. created the first synthesized graft with a layer of endothelial cells (Herring et al., 1979) by seeding the EC directly on a synthetic material, the Dacron®. However, the use of synthetic material still remains problematic because of the non-resorbability of the graft over time. Therefore research had continue to create fully biological TEBVs.

In 1998, L'Heureux et al. developed the first biodegradable TEBV (L’Heureux et al., 1998). The authors created the first TEBV based exclusively on the use of cultured human cells, without synthetic or exogenous biological material. This approach allowed the fabrication of three layers using fibroblasts to secrete an extracellular matrix on which SMC and endothelial cells were seeded. Mechanical tests revealed a mechanical strength of 2,600 mmHg. Unfortunately, the TEBV demonstrated a relatively short lifetime of 6 days, not compatible with implantation.

### 6.2 Traditional Biofabrication Techniques

One of the challenges in vascular tissue engineering is to find a method to distribute, organize and mature the different cells within the tubular structure at a large scale. To cope with this challenge, several methods have been developed, namely cell sheet engineering, decellularization of vascular tissue from a xenogeneic source and synthesis of scaffolds through electrospinning or molding, followed by cell seeding.

#### 6.2.1 Scaffold Cell Seeding Strategies

Scaffold-based strategies hold great promise for tissue engineering and regenerative medicine due to the existent 3D porous architectures for cell ingrowth and nutrients diffusion which facilitate tissue neo-formation and material biodegradation (Hutmacher, 2000; Yang et al., 2001; Hollister, 2005; Rezwan et al., 2006; O’Brien, 2011; Bose et al., 2012; Wu et al., 2014; Ovsianikov et al., 2018). This technique is based on seeding cells directly onto a scaffold, whose biocompatible structure will allow the cells to adhere and grow. The seeding can be static or dynamic through the use of a rotational systems (Villalona et al., 2010; Shamsah et al., 2020) but in every case, the scaffolds are immersed in different solutions containing the cells of interest until these latter adhere to the scaffold.

**FIGURE 3:**
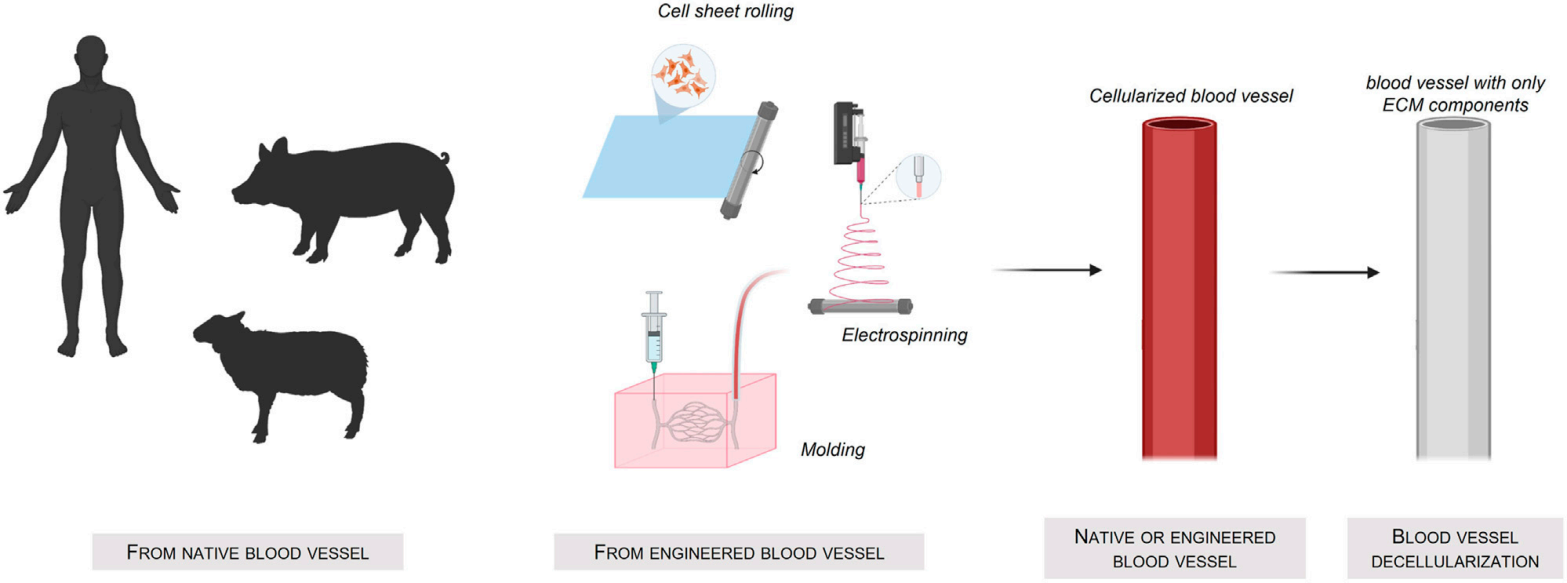
Fabrication of tissue-engineered blood-vessel using a native or engineered blood vessel source.

A very straightforward way to obtain interesting scaffolds is to use decellularized xenogeneic tissues (Figure 3) Created with Biorender®. The advantage of this approach is the preservation of the vascular architecture and the extracellular matrix layers complex composition. Nevertheless, despite the high availability of these xenogeneic tissues compared to human tissues, their use might be hindered by the possible severe immune responses (Ayala et al., 2018) of the host.

**FIGURE 4:**
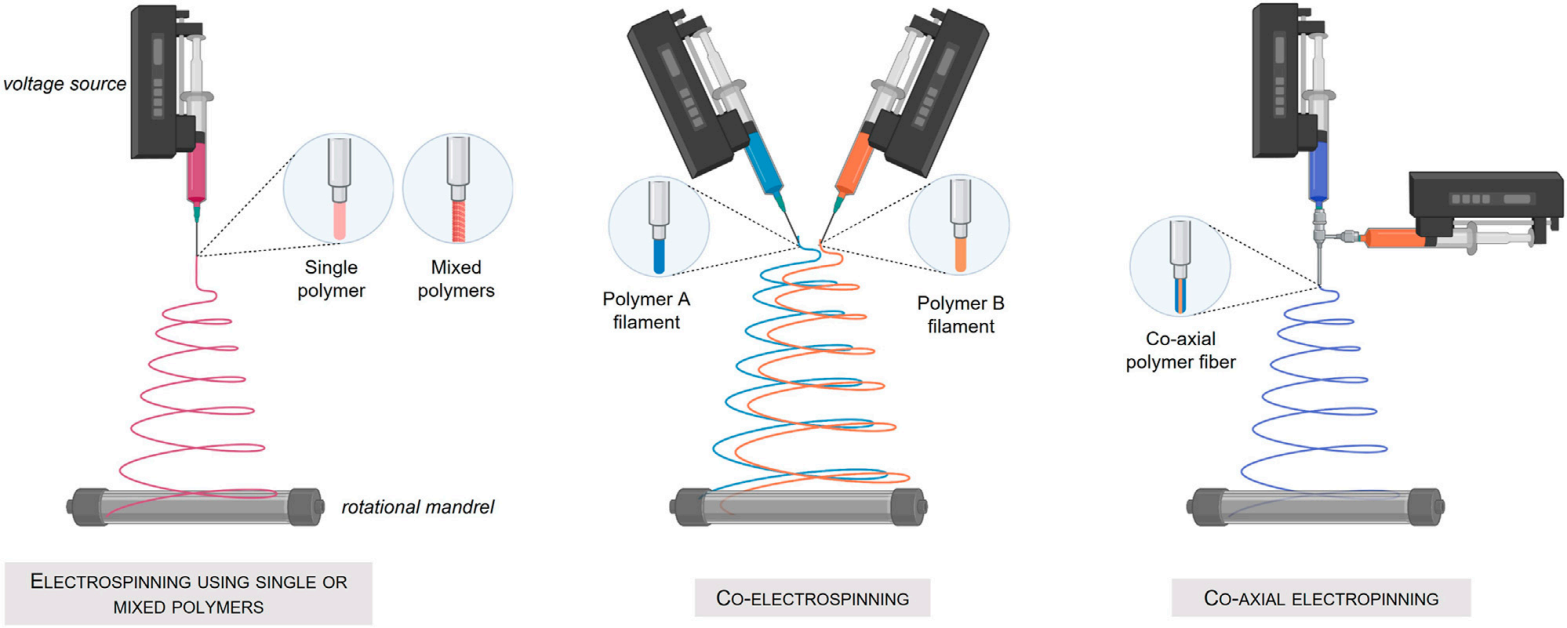
Fabrication of tissue engineered blood-vessel by the electrospinning technique. **(A)** Electrospinning using a single or mixed polymer. **(B)** Co-electrospinning of two different polymers. **(C)** Co-axial electrospinning.

An interesting alternative to produce porous scaffolds is the electrospinning process, a method using electrical force to form dissolved or melted down polymer fibers down to diameters of hundreds of nanometers. The deposited nanofibers are then capable of forming a highly porous mesh. In addition, electrospinning allows the simultaneous production of fibers of several different materials (Ramakrishna et al., 2006) and permits, when applied to a rotating support, the fabrication of tubular structures composed of three different layers. Nevertheless, this technique using high voltages and most of the time solvent-based material, cannot be applied to the direct deposition of living cells (Figure 4) Created with Biorender®.

Once a scaffold obtained, cells seeding become the most critical step in achieving recapitulated functional vascular systems. Cell density, homogeneity and integrity are key factors for vascular tissue seeding and then functionality (Vunjak-Novakovic et al., 1998). Passive seeding is up to now the standard procedure but is coming with drawbacks such as heterogeneous distribution of cells in the scaffold, high dependency upon cell migration within the scaffold and porosity of the biomaterial used to make the scaffold (Pawlowski et al., 2004) To overcome the disadvantages of static seeding, dynamic seeding techniques have been developed, in particular through the use of rotational systems (Godbey et al., 2004). This technique uses centrifugal forces that facilitates the homogeneous transfer of cells into porous scaffolds. A special rotor has been designed to fit into a conventional high-speed centrifuge such that the axis of rotation passes through the central lumen of the cylindrical scaffold. Human bladder SMC suspension was for example spun at 2,500 rpm into porous PGA scaffolds. The authors reported a greater seeding efficiency and homogeneity when compared to static or spinner flask techniques. Nevertheless, the authors also noted that the technique has a negative, although not quantified, impact on cellular viability and morphology.

An alternative seeding procedure was developed using magnetic fields. This technique is based on the guidance of magnetically labeled cells placed within a magnetic field gradient, leading to the cell seeding into tubular structures (Perea et al., 2006). To do so, an electromagnet surrounding a tubular collagen scaffold attracts the cells from the internal tubular structure through the collagen membrane walls of the tube, where they will be seeded and adhere. This action is reproduced with different cell types until the different vascular layers are obtained.

Last but not least, the *in vivo* scaffold cellularization and cultivation is an interesting alternative. This method allows the integration of the structure directly into the host system, enabling a rapid cellularization (Zhang et al., 2008; Nieponice et al., 2010; O’Brien, 2011; Loh and Choong, 2013; Thottappillil and Nair, 2015; Wu et al., 2018). However, the complexity of the scaffold interaction with the host cells (circulating, adherent, immune etc.) makes the prediction of the scaffold fate a difficult task, often resulting in fibrosis and thrombosis phenomena, obstruction of the pores and finally a slow degradation of the scaffold (Abbott et al., 1987; Tucker et al., 2009; Tatterton et al., 2012).

#### 6.2.2 Molding Cellularized Biomaterials

**FIGURE 5:**
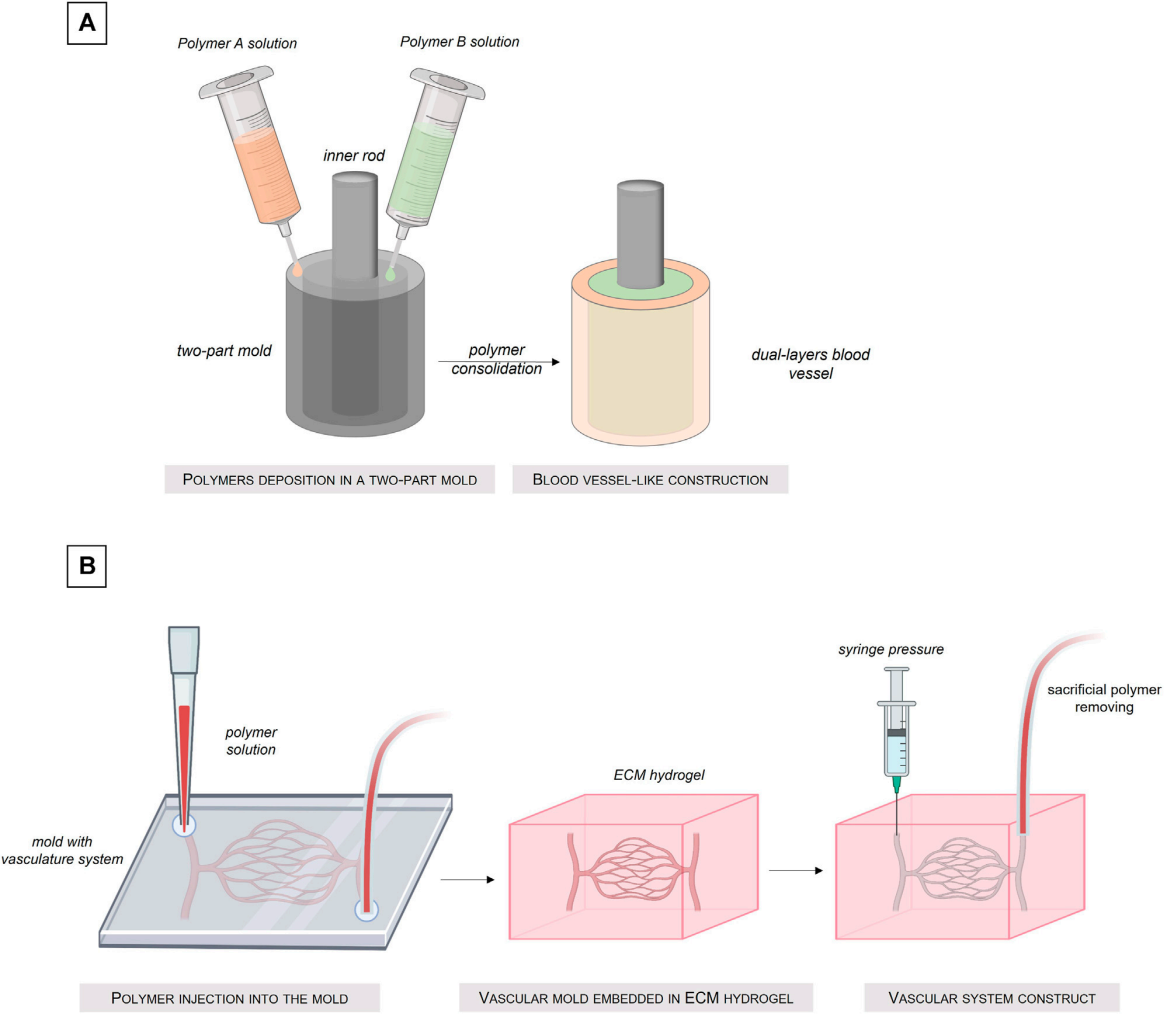
Fabrication of tissue-engineered blood vessel by the molding technique. **(A)** Injection of different polymer solutions into a dual-compartments mold. **(B)** Sacrificial molding intro an ECM hydrogel.

Molding cellularized biomaterial allows the manufacture of custom shape structures (Hasan et al., 2015; Kinoshita et al., 2016; Seifu et al., 2018; Santos-Rosales et al., 2020). This method is based on a simple process: a biomaterial solution is simply poured into a mold with the external shape of the desired structure. Once the biomaterial solution solidified, reticulated or gelled, the mold can be removed and the obtained structure released (Figure 5) Created with Biorender®. The main advantage of this technique is that a large range of shapes is possible, by simply changing the architecture of the mold.

In their work, Florian Helms et al. realized a trilayered blood vessel using the step by step casting technique (Helms et al., 2021). In a first step, ASCs were differentiated into SMCs which were integrated into a tubular fibrin matrix compacted in a mold to create the media layer. In a second step, a lower concentration fibrin matrix, mimicking the adventice layer, was molded around the media layer. Finally, luminal seeding with HUVEC was performed to cellularize the intima layer. Even if the final structure recapitulates finely the three layers organization of the blood vessel, a non-homogeneous seeding of cells on the molded structure was evidenced by the authors because of the static process of this method. Indeed, the cells contained in the liquid fibrinogen solution until it polymerizes, are subjected to the force of gravity and sediment at the bottom of the mold. In addition, this method involves the HUVECs seeding in the internal part of the tube and as we have seen previously, this method does not generate an homogeneous adhesion of the cells to the walls of the tube if no rotational system is perform for a long period of time.

Another molding vascular construct was developed by Keita Kinoshita et al. In their work, the authors had fabricated multi-layered vascular tissue models by cell layer deposition in an agarose hydrogel mold. The SMCs were embedded in the agarose mold and the EC were seed in the lumen of the vascular construct. In comparison with the previous method, the authors had the possibility to manufacture vascular construct with complex shapes, but the problem of cells non-homogeneity remains. In addition, the molding technique requires in the majority of cases a step of gelation of the cell solution mixed with a solution of biomaterial to obtain a three-dimensional construct. However, the crosslinking time, depending on the biomaterials, is not instantaneous and the homogeneity of the gelation of the construct requires correct diffusion of the solution inside the biomaterial. All these factors have an influence on homogeneity of the final distribution of the cells within the 3D construct.

#### 6.2.3 Cell Sheet Engineering

**FIGURE 6:**
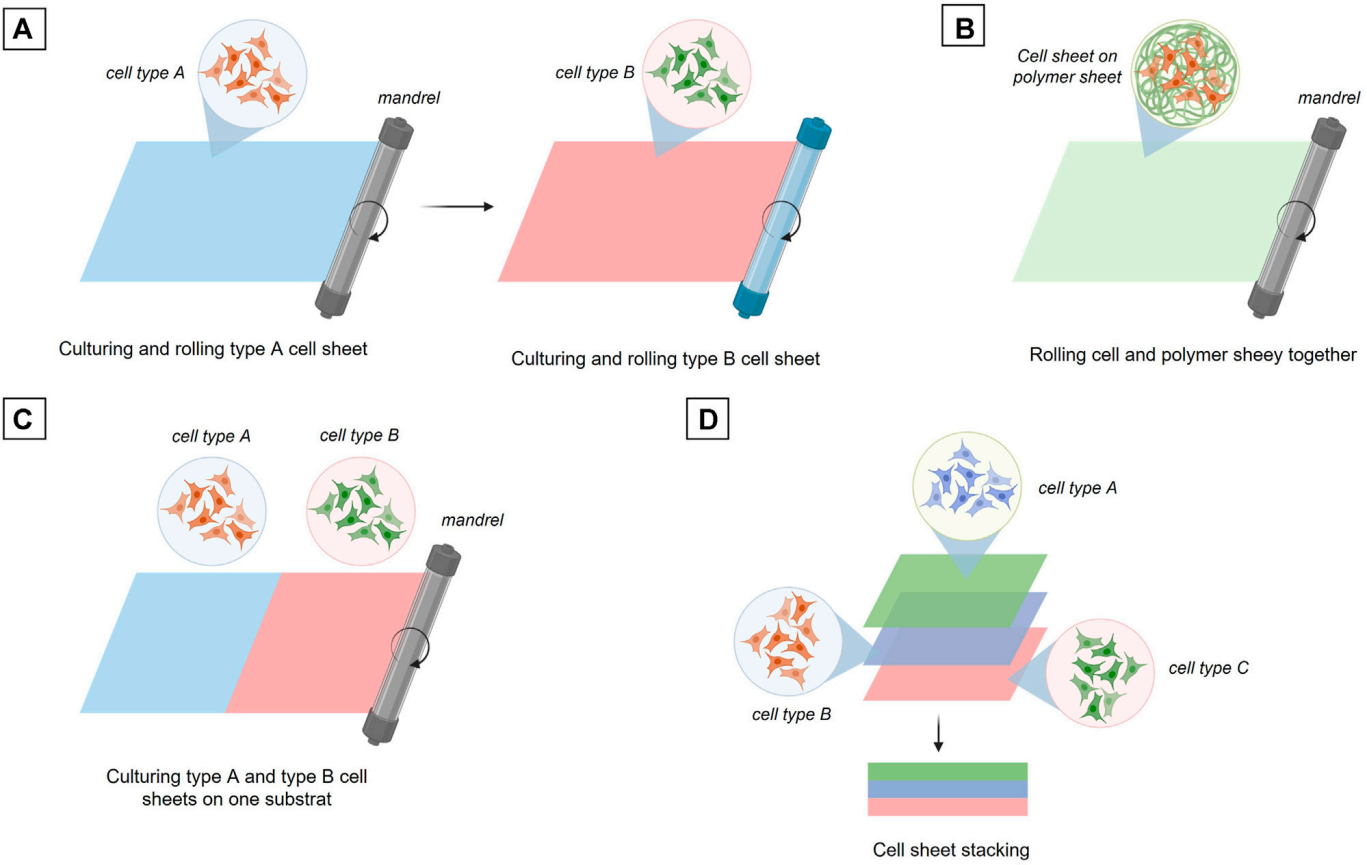
Fabrication of tissue engineered blood vessel through cell sheet engineering. **(A)** Sequential rolling of different cell type sheets. **(B)** Rolling of a culturing cell sheet onto a polymer sheet. **(C)** Rolling two different cell types culturing on the same substrat. **(D)** Three cell sheets stacking.

The cell sheet rolling technique is based on the direct use of cell layers cultivated in 2D up to confluency and then released from the culture surface or transferred with their 2D substrate (Yamato and Okano, 2004). The standard principle of this method is to wrap a first cell layer around a tube, a mandrel, then wrap a second layer composed of a different cell type and continue this process until the final construct is obtained, i.e. a vascular tube composed of the desired number of cell layers (Figure 6A) Created with Biorender®. As an alternative, the different cell types can also be co-cultured in 2D on the same substrate and then wrapped together around a mandrel (Figure 6B). Another possibility is that a biomaterial coating layer of a few millimeters can be created and used as a support for the 2D cultured cells. The cells layer onto the biomaterial layer is then rolled up around the mandrel. This cell sheet technique usually allows a higher mechanical strength of the final vascular tube depending on the biomaterial used as a support for 2D cell growth (Figure 6C).

The last technique to date using cell sheet is the cell sheet stacking method which allows the different cell layers to be assembled one on top of each other (Figure 6D). This principle makes it possible to study the interactions between the different vascular layers more easily and rapidly without the manufacturing process of a multi-tubular structure, which remains a complex process (L’Heureux et al., 1998; Ratcliffe, 2000; Konig et al., 2009; Gauvin et al., 2010; Kumar et al., 2013; Catto et al., 2014; Othman et al., 2015; Holland et al., 2018).

All these fabrication techniques enabled the creation of vascular structures recapitulating as much as possible the native vascular structures. However, none of these methods allows a precise positioning of the cells in a 3D architecture because almost all of them are based on cell seeding. Moreover, these different techniques involved multi-steps, long and not straightforward processes, especially for the sequential fabrication of the three different vascular layers, where a maturation time is necessary between each cell type seeding step. To overcome these obstacles, tissue-engineering researchers are looking into the use of innovative advanced technologies such as additive manufacturing methods, for example the 3D bioprinting, enabling the production of trilayered cellularized systems, in a single fabrication step.

### 6.3 Advanced Biofabrication Technique: 3D Bioprinting

3D printing, or additive manufacturing, allows the creation of three-dimensional objects by depositing successive layers of material guided by computer-aided design (CAD) models.

When cells are added to the deposition material, the obtained formulation is called a bioink. The creation of these bioinks makes it possible to produce centimeter-sized 3D objects, with spatial control of the cell’s deposition within the object and without the absolute requirement of cell seeding (Mironov et al., 2009; Visconti et al., 2010; Murphy and Atala, 2014; Mandrycky et al., 2016; Lee et al., 2018). Thus, the so called bioprinting technique allows the creation of complex and functional heterocellular structures having the potential of anatomical morphology and precision cell’s deposition (Datta et al., 2017).

Today, many techniques exist enabling bioprinting: inkjet drop deposition (Iwanaga et al., 2015), laser-assisted deposition (Koch et al., 2013), stereolithography (Melchels et al., 2010) and microextrusion (Ning and Chen, 2017). However, not all of them are suitable for the production of vascular tissue, in particular for the formation of vascular tissue composed of three different cell layers of centimeter-size tubular architectures.

**FIGURE 7:**
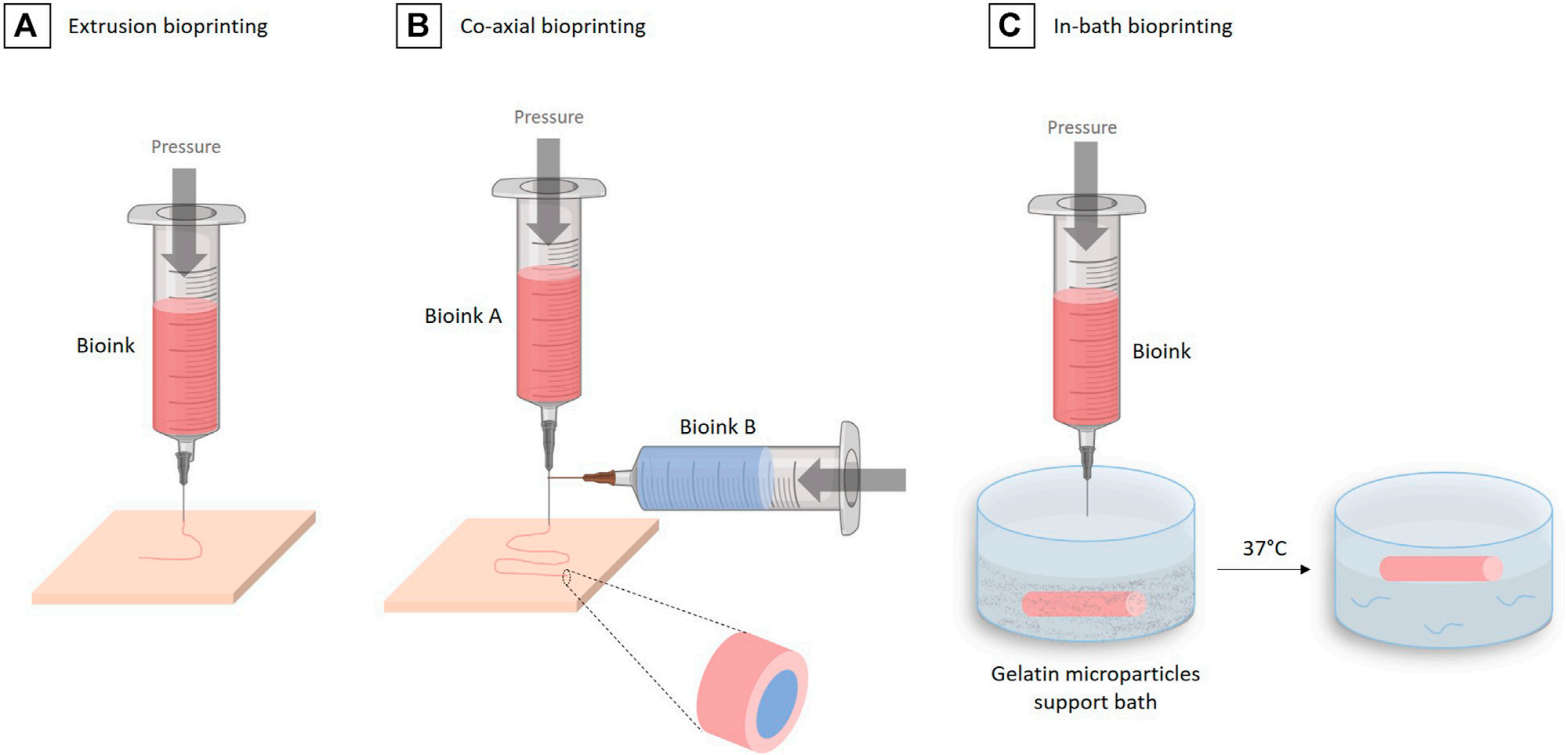
The different 3D bioprinting techniques for vascular tissue engineering. **(A)** Extrusion bioprinting. **(B)** Co-axial bioprinting. **(C)** In-bath bioprinting.

The most suitable technology for the creation of such large vascular tissue is microextrusion based-bioprinting (Ozbolat and Hospodiuk, 2016) in which a bioink, containing the appropriate biomaterials and cells, is loaded into a printing cartridge and extruded through a nozzle via a mechanical or pneumatic system (Figure 7A) Created with Biorender®. The advantage of the microextrusion-based bioprinting is the wide choice of possible biomaterials and cells that can be used (Table 1), helping recapitulate the complexity of the vascular tissue structure. Moreover, multiple microextrusion heads bioprinters are now available on the market (CELLINK®, FELIX® printers, RegenHu®, Allevi3D®, BioAssemblyBot®), allowing the use of several extruders at the same time to further complexify the cell constructs.

One technique in particular allows the easy and fast reconstruction of multiple-layered vascular tissue: the coaxial technique (Jia et al., 2016; Pi et al., 2018; Gao et al., 2019), which is based on the use of special coaxial nozzles, i.e. a nozzle within a nozzle (Figure 7B). This method allows to “wrap” one bioink in another. This approach makes sense in vascular engineering where one wants to form several concentric layers, while forming a tubular structure.

In their work, Ge Gao et al. used such a triple coaxial bioprinting technique to fabricate a biomimetic TEBV by using two different bioinks, composed of 3% (w/v) vascular-tissue-derived extracellular matrix (VdECM) and 2% (w/v) alginate, mixed with VSMCs or ECs, to form the *media* and the *intima* layers, respectively. After the co-axial bioprinting and consolidation using a 100 mM CaCl_2_ solution, the construct maintained its 3D shape during the maturation phase. After a 3 days of static culture in a 37°C, 5% CO_2_, incubator and 2 weeks maturation under pulsatile stimulation, constructs were fixed for histological analysis. The staining of VE-cadherin (an intercellular junction protein between ECs) and α-SMA (an actin isoform represent contractility of VSMCs) revealed that the coaxially printed constructs exhibited an endothelialization rate of 96%. The authors also investigated the TEBV performances during an *in vivo* study in a rat model. The TEBV implanted was wrap into a PCL (polycaprolactone) sheet to prevent its rupture. The implant was maintained *in vivo* during 3 weeks and was shown to conserve an open lumen and its permeability (Gao et al., 2019).

In 2020, the same group further developed the technique of “in-bath triple coaxial cell printing” (Gao et al., 2020), in which they used the same method as in their previous work, but printed directly in a pre-gel bath containing fibroblasts. The adventitia layer, missing in their first work, was then formed by this fibroblasts gel surrounding the tube extruded using the coaxial technique (Figure 7C).

This coaxial technique remains to date the most advanced method to create a vascular tissue with the expected trilayered structure. However, the size and architecture of the obtained blood vessel is limited by the size of the coaxial nozzle. A technology allowing to bioprint each layer independently but in the same construct and in the shortest possible time, remains then to be demonstrated.

## 7 Tissue Maturation

The manufacture of the three vascular layers, composed of endothelial cells, smooth muscle cells and fibroblasts, is not enough to obtain a functional blood vessel. The next phase, called maturation, is perhaps the most complex, critical and longest phase in the creation of functional vascular tissue. During this maturation step, cells will be able to adapt to their 3D environment, produce an extracellular matrix and establish cell-cell connections (Mertsching and Hansmann, 2009).

Numerous studies have shown that cell and tissue maturation is increased in response to mechanical stresses under fluid flow (Kobayashi et al., 2004; Ruan et al., 2015; Ruan et al., 2016; Homan et al., 2019). In addition, cell maturation, until a tissue is created, is also a reflection of an *in vivo*-like environment. This is why the use of bioreactors for tissue engineering has become a real challenge.

For several years, the main objective of the use of bioreactors in the field of vascular engineering has been the endothelialization of the internal layer of a scaffold (Çelebi-Saltik et al., 2019). Today, fine and precise control of cell maturation and proliferation inside the construct becoming vital for obtaining tissue, the use of specific bioreactors has become necessary.

### 7.1 Bioreactor Functions

In the blood vessel field, bioreactors can be classified into three categories: static bioreactor, dynamic bioreactor and biomimetic bioreactor. A static bioreactor enables a passive maturation of the construct, in classical culture vessels placed in a controlled atmosphere incubator. Dynamic bioreactors are composed of a culture vessel equipped with an inlet and an outlet, making it possible either to perfuse endothelial cells inside a construct for endothelialization or to perfuse a flow inside a pre-cellularized construct, inducing then cell maturation through hydrodynamic stress (Park et al., 2012; Bono et al., 2017; Lin and Mequanint, 2017; Wang J. et al., 2017; Kural et al., 2018; Pennings et al., 2019; Pennings et al., 2020; Çelebi-Saltik et al., 2021). Finally, biomimetic bioreactors are designed to recapitulate the *in vivo* tissue environmental conditions. The system allows the control of multiple parameters such as perfusion flows for physiological stimulation of the cultured cells, but also culture conditions such as pH, temperature, oxygen and medium culture feeding. The key objective of these biomimetic bioreactors is to provide a sufficient supply of nutrients and oxygen while removing waste products to allow a correct cell proliferation and viability inside the tissue while mimicking *in vivo* shear stress conditions to allow vascular cells to accurately recapitulate their physiological functions (Martin et al., 2004; Yu et al., 2004; Griffith and Swartz, 2006; Lujan et al., 2011; Hansmann et al., 2013).

### 7.2 Bioreactors Design

**FIGURE 8:**
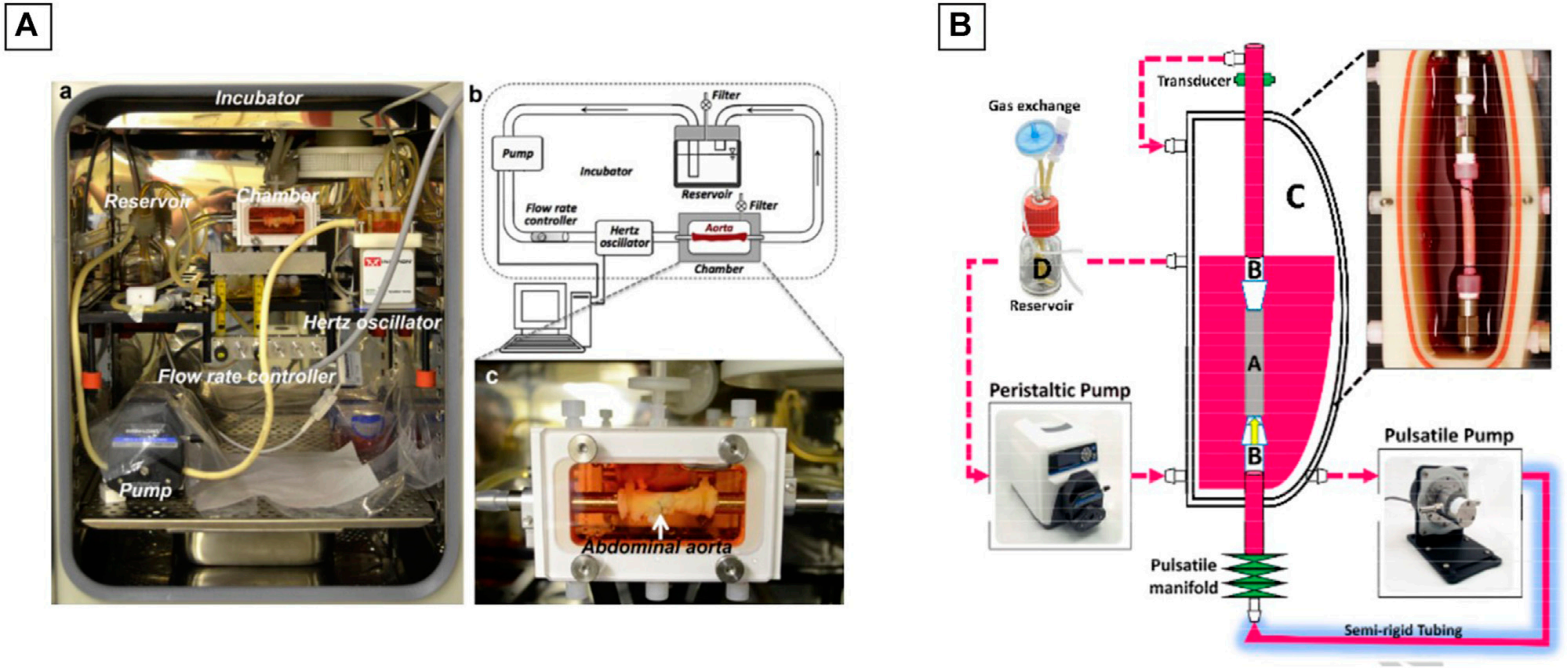
Commercial bioreactors conception. **(A)** The LumeGen biorector design; **(B)** The ElectroForce® biorector design. The image is adapted from the work of Wang J. et al. (2017) and Lin and Mequanint (2017).

Juan Wang et al. used the commercially available LumeGen bioreactor (Wang J. et al., 2017) for their work. This bioreactor was used to provide a controlled environment for simulating physiological aortic conditions. The bioreactor consisted of a blood-vessel chamber, a variable-flow pump, a hertz oscillator, a flow control channel and a culture medium reservoir, all placed within a standard incubator (37°C and 5% CO_2_) (Figure 8A). A bioreactor commercialized by ElectroForce® was also used during the work of Shigang Lin and Kibret Mequanint (Lin and Mequanint, 2017) for the maturation of a functional blood vessel. The bioreactor system consists of five principals components: 1) a culture medium reservoir equipped with a filter for gas exchange, 2) a pulsatile pump with sterilizable pump head, 3) a sterilizable BioDynamic® chamber, 4) a pulsatile manifold to generate luminal pulsation and 5) a pressure transducer, if pressure measurement is needed (Figure 8B).

**FIGURE 9:**
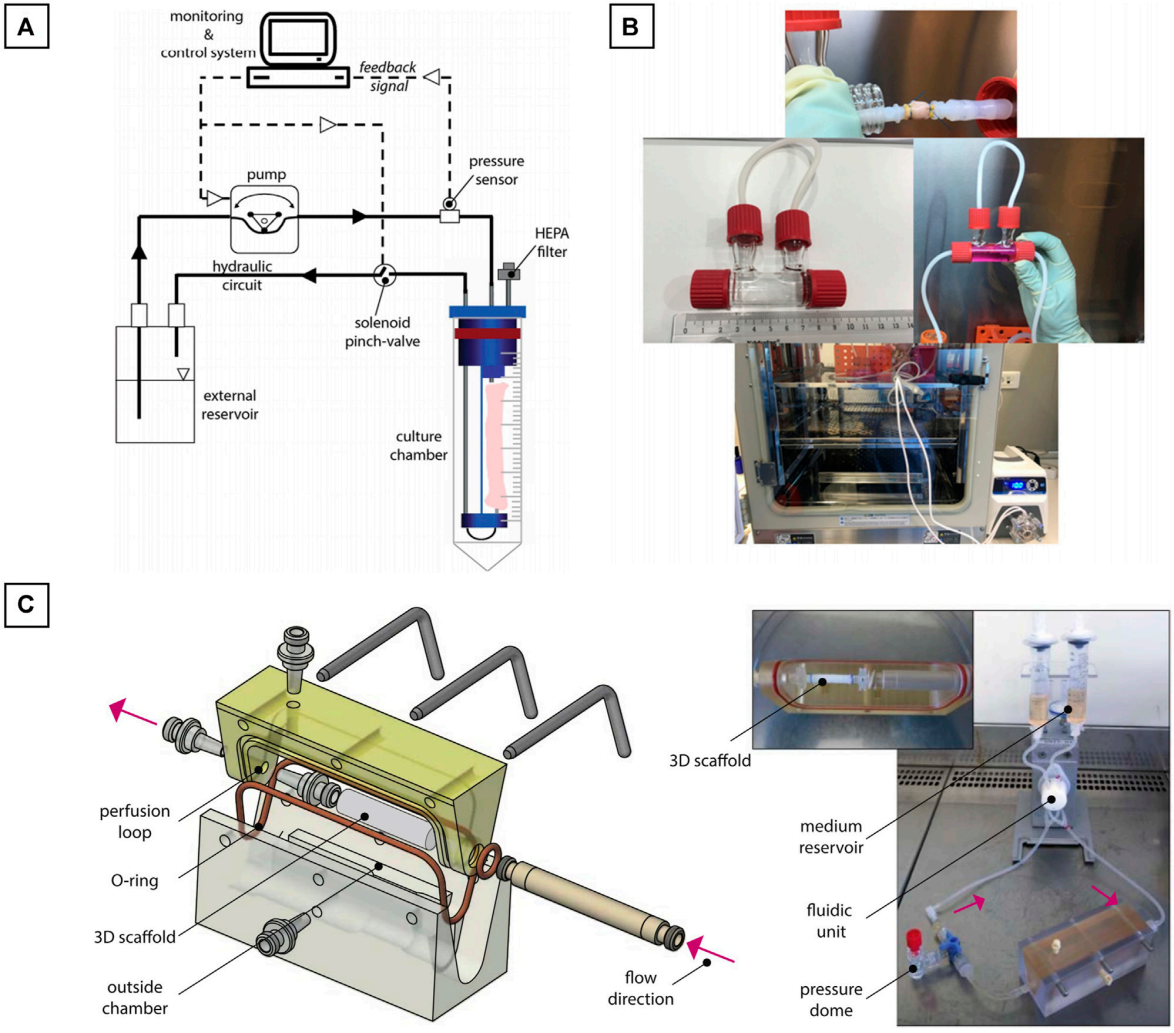
Homemade bioreactors ideas. **(A)** Dual-mode bioreactor system; **(B)** Perfused bioreactor and **(C)** Perfused biorector for a bi-layered vascular graft. The image is adapted from the work of Bono et al. (2017), Çelebi-Saltik et al. (2021), and Pennings et al. (2020).

However, a large majority of the bioreactors used in vascular engineering are homemade, having the advantage of being easily customized. In their work, Bono et al. created a dual-mode bioreactor system (Bono et al., 2017) (Figure 9A), useful for the fabrication of the vascular construct (construct mode) and for the *in vitro* stimulation of the vascular construct (culture mode). Betül Çelebi-Saltik et al. designed also their own homemade perfused bioreactor (Çelebi-Saltik et al., 2021). The system was composed of one chamber and was connected to a culture medium reservoir (Nalgene® Sterile PETG Media Bottles, Rochester, NY, United States), which had three ports that allowed fluid and air circulations. Silicone tubes acted as a bridge between the chamber and reservoir. Medium circulation was carried out with a pump in the inner lumen of the vessel (MasterFlex, Cole Parmer) at a flow rate of 10 ml/min This bioreactor was placed directly inside a classical at incubator (37°C and 5% CO_2_) (Figure 9B).

Iris Pennings et al. developed another homemade perfused bioreactor for a bi-layered vascular graft (Pennings et al., 2020). In their work, a two-compartment bioreactor was created. This system was able to apply a physiological shear rates at the luminal side of the vascular construct for the endothelial cells proliferation and at the same time to expose the outer layer to another culture medium. The bioreactor was composed of a custom-made culture chamber with the tubular construct inside. The luminal side of the construct was connected to a flow loop for unidirectional flow application, whereas the outer side of the scaffold was exposed to static culture conditions. The flow inside the construct was controlled by an Ibidi pressure pump (Ibidi GmbH, Martinsried, Germany). The entire system was placed directly inside a classical at incubator (37°C and 5% CO_2_) (Figure 9C).

### 7.3 Bioreactors Strategies for Maturation

In the field of vascular engineering, the choice of the bioreactor system depends on the expected maturation conditions of the vascular construct. Different strategies of maturation exist for blood vessel development: with a flow through the vessel (Bilodeau et al., 2005; Mcfetridge et al., 2007; Bjork and Tranquillo, 2009), with mechanical stimulation with a rotational bioreactor (Bilodeau et al., 2005; Blaber et al., 2010) or with burst pressure (Bilodeau et al., 2005; Hahn et al., 2007).

One of the first studies of bioreactors for vascular grafts was carried out in 1999 by Niklason et al. who demonstrated the vital contribution of a flow inducing shear stress for the migration of smooth muscle cells through a vascular structure scaffold (Niklason et al., 1999).

Jing Zhou et al. also demonstrated the importance of shear stress on endothelial cells (Zhou et al., 2014). This stress activates a signaling path that regulates the proliferation, maturation and quiescence of SMCs in the next layer. The shear stress induced by the flow within the bioreactor as well as the communication between the different cells present in the vascular tissue appears to be a crucial step in the formation of a functional vessel.

Some bioreactors also perform pulses to mimic heartbeat. These pulses caused by burst pressure allow stretching forces on the vascular wall and have an impact on cells maturation. In their study, Chen Wang et al. created a vascular construct placed under dynamic stimulation for 8 weeks (Wang et al., 2010). The vascular construct exhibited a dense and well-organized structure similar to the native vessel and exhibited a significant improvement in biomechanical properties when compared to control maturation under static conditions.

Today, bioreactors for vascular engineering are most of the time used for scaffold seeded with vascular cells in order to obtain a complete endothelialization and thus the formation of the intima layer only. A gap then still remains on the recapitulation and maturation of the two other layers within bioreactors.

As we have seen previously, techniques such as 3D bioprinting shall make it possible to recreate trilayered vascular tissues including endothelial cells, smooth muscle cells and fibroblasts. However, once produced, unlike the acellular scaffolds, the construct does not have sufficient mechanical properties to resist a flow and to be manipulated (Weinberg and Bell, 1986). To acquire these capacities, the cells inside this construct must proliferate and mature, allowing in parallel, a degradation of the biomaterials and a secretion of the extracellular matrix, finally resulting in a mature tissue. Here is the paradox, to be functional, a newly recreated blood vessel must mature thanks to a bioreactor to obtain its physiological properties and sufficient mechanical resistance. However, to allow its integration into a bioreactor, the immature blood vessel must be strong enough to resist internal flow and to be maintained in the bioreactor, throughout the maturation process. In summary, an immature blood vessel must be placed in a bioreactor to become functional, strong and easy to handle for *in vitro and in vivo* testing, but the immature blood vessel is too fragile to be placed in a bioreactor.

## 8 Clinical Trials

Using biodegradable polymer-based for vascular constructs production, Skin’oka et al. were the first in 2001 to carry out a successful clinical application of a TEBV composed of PolyLactic Acid (PLA) and PolyCaproLactone (PCL) synthetic polymer (Shin’oka et al., 2001). The researchers explanted autologous vascular cells from a peripheral vein and seeded these cells on the engineered TEBV. Their work has demonstrated the integration of the cellularized biodegradable scaffold mimicking a pulmonary artery in a child with pulmonary atheresis. The negative aspect of this graft was the rather long cell expansion time with a production period of 8 weeks. In a complementary study, the same group used autologous bone marrow mesenchymal stem cells seeded directly on the TEBV. The cellularized construct were implanted to 25 patients and demonstrated a permeability and patient’s survival 7 months after its implantation. The most common problem was graft stenosis which happen for 28% of the patients.

Today, many TEBV based on the use of decellularized ECM are commercially available: Artegraft® (bovine carotid artery), Procol® (bovine mesenteric vein), SynerGraft® (bovine urethra) and Cryovein® (human femoral vein). The clinical trials of these grafts are compared with those of conventional ePTFE grafts. In clinical studies of Procol® vascular graft, researchers Katzman et al. found a permeability of this decellularized TEBV equivalent to that of a standard ePTFE graft (35 versus 28% at 12 months) (Katzman et al., 2005). However the Procol® graft was shown to reduce complication rate and morbidity. The decellularized TEBV SynerGraft®, Chemla and Morsy have demonstrated that the material has a lower permeability rate than that of the standard graft in ePTFE (28% against 48%) as well as a similar complication occurrence (96% against 91%) (Chemla and Morsy, 2008). Recently, the decellularized Humacyte® vascular graft has conducted its phase II clinical trials in patients with end-stage renal disease (Lawson et al., 2016). This TEBV is made from a PolyGlycolic Acid (PGA) scaffold on which smooth muscle cells from deceased organ and tissue donors are seeded, cultured until enough ECM is synthesized. At that point, the TEBV is decellularized and ready for implantation. The Humacyte® graft demonstrated a permeability of 63% at 6 months after implantation in 60 patients. This new Humacyte® vascular graft was also characterized by the absence of immune response and by a lower infection rate than for standard ePTFE grafts. However, at 12 months after implantation, the observed permeability rate of 18% is lower than for a standard ePTFE graft.

This combined approach to the use of a biodegradable polymer with decellularized ECM was initiated by Niklason’s group (Lawson et al., 2016). They conducted a clinical phase 2 trial on the use of a PGA graft seeded with SMC derived from organ donors and cultured for 8 weeks. The seeded SMC TEBV was then decellularized to remove allogeneic antigens. Autologous endothelial cells were seeded inside the TEBV 7 days prior implantation. These grafts were implanted in 60 patients with end-stage renal disease. No immune complications were observed and the achieved permeability compared well with ePTFE graft. After histological analysis of graft segments, luminal endothelialization and repopulation of vascular SMCs in the walls of the graft were observed. Phase 3 clinical trials are underway.

It was between 2004 and 2007 that L’Heureux et al. carried out the first clinical trial of cell sheets (L’Heureux et al., 2007). This graft consisted of three layers, a first one derived from autologous fibroblasts, an acellular middle layer and an inner layer of autologous endothelial cells. The process of creating this TEBV took between 6 and 9 months. These TEBV were transplanted on 10 patients with end-stage renal disease. Out of 10 patients who received this graft, three were subject to a failure of the integration, on patient was hemorrhaged and one deceased. The graft worked for 6–20 months in the remaining five patients with a permeability of 60% 6 months after implantation.

In their next clinical trial, L’Heureux et al. used their biofabrication method of sheets of allogeneic fibroblasts (L’Heureux et al., 2006a). Sheets of fibroblasts were grown for 8 weeks, then wrapped around a mandrel. For another 10 weeks, these fibroblast cell sheets of were grown and then dehydrated and stored at −80°C for 6–9 months. These grafts, called Lifeline®, were rehydrated a few days before implantation. The group carried out two case studies: one using grafts seeded with autologous endothelial cells (*n* = 1) and a second without seeding endothelial cells (*n* = 3). No immune response was detected in both study cases. However, in the second case of study, without endothelial cells seeding, thrombogenic failures were observed 3–5 months after implantation in two of the three grafts. Studies are underway to demonstrate the usefulness of allogeneic fibroblasts and to demonstrate the need to use endothelial cells to help avoid thrombogenic failures.

More recent studies have also demonstrated the usefulness of adding endothelial cells to TEBV as a solution to achieve more durable graft permeability. In their work, McIlhenny et al. generated endothelial cells transfected with an adenoviral vector carrying the endothelial nitric oxide synthase (eNOS) gene (McIlhenny et al., 2015). Then, these ECs were seeded on a decellularized human saphenous vein scaffold. According to their study, the synthesis of NO would prevent thrombotic occlusion. These grafts were implanted in five patients and demonstrated permeability with a non-thrombogenic surface 2 months after implantation of the graft.

Several techniques for manufacturing TEBV had led to their use in clinical trials. However, the advantages of these TEBV over vascular grafts have not yet been demonstrated and stenosis currently remains the main cause of failure. This complication was proven to be related to a gap of rigidity/elasticity between the graft and the patient’s native blood vessel. This property is mainly governed by the extracellular matrix of the blood vessels produced by fibroblasts and vascular smooth muscle cells. In particular, the synthesis of elastic fibers by fibroblasts allows the formation of a more flexible ECM, thus mimicking the physiology of native blood vessels. However, from all the models we have described above, none of them allow sufficient production of elastic fibers and this is a remaining challenge for the field.

## 9 Future Directions

Vascularization is one of the major factors allowing the viability of an artificially constructed tissue/organ thanks to the supply of oxygen and nutrients to the cells and through the elimination of waste. Since the research of Weinberg et al. who created the first TEBV formed from a collagen scaffold and seeded with endothelial cells, smooth muscle cells and fibroblasts, vascular tissue engineering made tremendous progress.

However, these technological progresses still do not make it possible to produce vascular tissue for all the patient demand (Roth et al., 2020). As a seldom example, the Cytograft’s Lifeline® vascular graft is a living conduit with the properties of a native vessel, constructed through the use of patient cells (L’Heureux et al., 2006b)*.* This vascular graft is one of the few not having any synthetic or exogenous material in its composition. However, the production time of this Cytograft’s Lifeline® vascular graft is still between 6 and 9 months with most of the production dedicated to the fibroblast expansion and growth until sufficient collagen is produced.

The use of allogenic adult stem cells seems to be one solution to this issue. As these cells grow rapidly, they would provide tissue within 10 weeks without using cells from patients (Bajpai and Andreadis, 2012; Hirschi et al., 2014; Gui et al., 2016; Wang Y. et al., 2017; Chan et al., 2018; Klein, 2018; Gorecka et al., 2020). However, more basic molecular research is requested to fully understand the advantages and disadvantages of using this cellular source.

To go further in the creation of biomimetic vascular tissues, research in tissue engineering must be directed towards the phenotypic heterogeneity of cells according to the organs to be reproduced. Endothelial cells do not have the same phenotype depending on the organ or depending on the area of the organ. This point was particularly clearly demonstrated in the case of the liver (Rafii et al., 2016; Géraud et al., 2017; Marcu et al., 2018). This is the same for smooth muscle cells whose differentiation depends on their location and function and is governed by molecular pathways (Yoshida and Owens, 2005; Deng et al., 2006; Owens, 2007). Understanding the developmental pathways determining embryologic differentiation into organ-specific cells may suggest potential strategies for stem cell engineering.

Today, in a vast majority of studies, vascular constructs are composed of biomaterials with only one layer of endothelial cells. However, real blood vessels should not only contain endothelial cells but also smooth muscle cells which are vital for ensuring permeability and mechanical strength, but also fibroblasts to recreate the extracellular matrix specific to a fully mature vascular tissue. Current blood vessel manufacturing with different techniques such as cell sheet rolling, cell molding, electrospinning, have reach limits: lack of homogeneity in cell distribution, poor mechanical strength of the construct, limited lifespan, limitation in exact representation of the three cellular vascular layers in cylindrical form. The production of vascular tissues, therefore, remains a challenge for tissue engineering. 3D bioprinting then arises as a promising technology to meet this challenge. Using this advanced technology, researchers can reproduce desired anatomical models with great precision. In addition, thanks to the formulation of specific biocompatible bioink, cells can organize themselves in 3D, proliferate, migrate and differentiate efficiently.

However, the mechanical rigidity of the matrix obtained after the bioprinting is dependent and has an impact on stem cell differentiation. It will therefore be important to carry out more in-depth studies to link bioink compositions to differentiation phenomena.

The ideal technique for recreating the structural shape of a blood vessel with its three layers of different composition and concentrically organized, is still to be unveiled. Electrospinning, cell sheet rolling and even molding techniques make it possible to create tubular layers one after the other. The disadvantage of these techniques is therefore that they take several hours, or even days, to complete. In addition, in these methods, the creation of each layer is dependent on the formation of the previous one. With the arrival of 3D bioprinting technology, notably thanks to new 3D printers composed of several print heads, the creation of a multi-material structure, quickly and with personalized shape, becomes possible. However, the different studies in 3D bioprinting have not yet demonstrated their capacity to reproduce, in a single step, the three different layers of a blood vessel. The concentric bioprinting of endothelial, smooth muscle and fibroblast cells is therefore expected to be an important area of investigation for the future.

Finally, an essential parameter to take into account is the immune response of the host during the implantation of the reproduced blood vessel. Again, further studies on stem cells are needed to better understand their behavior. Also, more in-depth research on “biomaterials-blood” contact interaction as well as the degradation of these biomaterials after implantation, need to be deepened. This is particularly the case for vascular tissue produced *in vitro* from biomaterials and heterologous human cells, which could elicit immune responses in the patient.

## 10 Conclusion

After many years of research in the field of vascular tissue engineering, obstacles remain. Although 3D bioprinting has enabled a breakthrough since the creation of the first TEBV over half a century ago, more work is required in the fabrication of clinically relevant vascular constructs. The complexity of the developed constructs must be improved, as well as the composition of the biomaterials associated with the different cell types. In addition, advances in the cell source, biomaterials formulation, manufacturing technologies and maturation are crucial and a source of motivation for the world of tissue engineering. Once these challenges addressed, huge progresses are expected in the realization of functional trilayered blood vessel.
